# Would you fund this movie? A reply to Fox et al. (2014)

**DOI:** 10.3389/fpsyg.2014.01428

**Published:** 2014-12-09

**Authors:** Timothy D. Wilson, Daniel T. Gilbert, David A. Reinhard, Erin C. Westgate, Casey L. Brown

**Affiliations:** ^1^Department of Psychology, University of VirginiaCharlottesville, VA, USA; ^2^Department of Psychology, Harvard UniversityCambridge, MA, USA; ^3^Department of Psychology, University of California, BerkeleyBerkeley, CA, USA

**Keywords:** thinking, directed thought, consciousness, affect, enjoyment, boredom

We thank Fox et al. (2014) for their interest in our research and welcome this opportunity to respond to their commentary. They argue that participants in our studies enjoyed “just thinking” more than we claimed (Wilson et al., 2014). We found some irony in their position, because we began this line of research with a similar hypothesis. As the data came in we were surprised that participants did not enjoy deliberative thought very much, even when we went to some lengths to give them time to prepare and choose their topics (i.e., in our “prompted fantasy” conditions). We are thus in the rather amusing position of explaining why both Fox et al.'s interpretation and our initial hypotheses are wrong.

Our view can be summarized with a simple thought experiment. Imagine you are a venture capitalist, and a filmmaker comes to you asking for money to make a full-length feature film. You decide it would be wise to show the film maker's 15-min trailer to some focus groups. You find that (a) on average, the focus groups rate the trailer as much less enjoyable than a variety of everyday activities, such as listening to music and surfing the web; (b) between a third and a half of the people in the focus groups disobey the instructions to watch the trailer and instead prefer to do something else, such as playing with their cell phones; and (c) the majority of the men (and a quarter of the women) in the focus groups choose to give themselves an unpleasant electric shock while watching the trailer, even though they had said earlier that they would pay to avoid getting the shock. Would you invest? Probably not. These are, of course, precisely the results we found in our studies when we left people alone with their thoughts. As such, we do not think it is unreasonable to conclude that the participants found deliberative thought to be neither engaging nor enjoyable.

Although our space is limited, we will respond to a few of Fox et al.'s specific arguments. First, the results they report in Figures 1A–C are entirely consistent with the data we reported in our Table 1. Instead of quibbling over what it means for participants to rate something as somewhat enjoyable and somewhat boring, we believe it is more informative to compare the ratings of participants under different experimental conditions and to observe their actual behavior. In Study 8, participants rated everyday external activities as much more enjoyable than deliberate thinking (the distribution of responses in the two conditions was almost non-overlapping). Fox et al. suggest that we stacked the deck in favor of the external activities by letting people choose among activities that “were not banal or boring.” But note that participants in the “thinking” condition were completely free to think about anything they wished, to choose topics that were “tailored to their personal interests,” and to switch between topics. They could choose to think about past accomplishments, current interests, or future plans, or even, to build on Fox et al.'s analogy, chocolate and sex. The fact that participants much preferred the everyday external activities is quite telling, we think, and does not speak well for the appeal of “just thinking.”

We also find it revealing that 32–54% of participants (depending on the study) disobeyed instructions to “just think” and performed external activities they were specifically asked to avoid, such as using their cell phones or doing school work. (Note that these figures include only those who admitted to doing the banned activities.) If deliberative thinking is as enjoyable as Fox et al. suggest, why did so many participants disobey explicit instructions to do it for relatively short periods of time?

Fox et al. are unimpressed by the results of our study in which 67% of men and 25% of women administered at least one shock to themselves. Their argument that participants were simply curious is at odds with the fact that all participants had already experienced the shock and that we specifically selected only those participants who reported that they would pay to avoid another shock. Fox et al. argue that 14 of the 18 participants who shocked themselves reported that they did so out of “curiosity about (or interest in) the quality of the shock or its effects as their motivation.” But note that what many of these participants were interested in was how unpleasant the shock would be, such as the participant who wrote, “I wanted to see if the shock felt as painful the second time as it did the first time.” If thinking is so enjoyable, why would participants be willing to re-experience an unpleasant stimulus just to see if it is still unpleasant? (Not to mention the fact that participants' reports about the reasons for their behavior are, in large part, *post-hoc* rationalizations that may not reflect their actual reasons; Nisbett and Wilson, 1977; Johansson et al., 2005). Lastly, we note an error in one of Fox et al.'s statements. Participants did not rate their “enjoyment of ‘just thinking,”’ but rather their enjoyment of the thinking *period*, including any shocks they administered. Thus, if people shocked themselves because they were bored, and the shocks helped alleviate that boredom, it is not surprising that the shockers rated the overall experience similarly to the non-shockers.

More fundamentally, Fox et al. seem to have misunderstood what our studies were about. They described our research as having investigated “spontaneous thought,” which one of the authors defined as “the unintended, non-working, non-instrumental mental content that comes to mind unbidden and effortlessly” (Christoff, 2012, p. 52). But in fact we examined directed conscious thought, because in virtually all of our studies we specifically instructed participants to think in enjoyable ways. As Christoff (2012) noted, “It is impossible, after all, to instruct participants to have a spontaneous thought” (p. 57). No other studies, to our knowledge, have examined how successfully people can engage in deliberative thinking when the goal is enjoyment or positive affect. Fox et al.'s comparison of our research to previous studies of spontaneous thought is thus moot.

Clearly some kinds of thinking are enjoyable. As we said at the end of our article, “There is no doubt that people are sometimes absorbed by interesting ideas, exciting fantasies, and pleasant daydreams.” But we also noted that “it may be particularly hard to steer our thoughts in pleasant directions and keep them there” (p. 77). The preponderance of the evidence supports this conclusion, we believe, and is contrary to both Fox et al.'s and our own initial hypotheses.

## Conflict of interest statement

The authors declare that the research was conducted in the absence of any commercial or financial relationships that could be construed as a potential conflict of interest.
